# Supplementary material for: Caffeine and Cisplatin Effectively Targets the Metabolism of a Triple-Negative Breast Cancer Cell Line Assessed via Phasor-FLIM
Source: Int J Mol Sci. 2020 Apr 1;21(7):2443. doi: 10.3390/ijms21072443 (PMC7177700; doi:10.3390/ijms21072443)
Supplement: Supplementary file 1 [file ijms-21-02443-s001.pdf]

Supplementary Material

Caffeine and Cisplatin Effectively Targets the Metabolism of a Triple-Negative Breast Cancer Cell Line Assessed via Phasor-FLIM

Stephanie M. Pascua[1,†], Gabrielle E. McGahey[1,†], Ning Ma[3,†], Justin J. Wang[4] and Michelle A. Digman[1,2*]

[1] Department of Biomedical Engineering, University of California, Irvine, 92617, California, USA smpascua@uci.edu (S.M.P.), mcgaheyg@uci.edu (G.E.M.), mdigman@uci.edu (M.A.D.)
[2] Laboratory for Fluorescence Dynamics, University of California, Irvine, California, USA
[3] Department of Surgery, Stanford University School of Medicine, Stanford, CA, USA
[4] Department of Computer Science, Stanford, California, USA ; Jjwang01@stanford.edu (J.J.W)
† These authors contributed equally to this work.
* Correspondence: mdigman@uci.edu (M.A.D)

Figure S1. Fluorescence lifetime trajectories of the metabolic state of MDA-MB231, MCF7

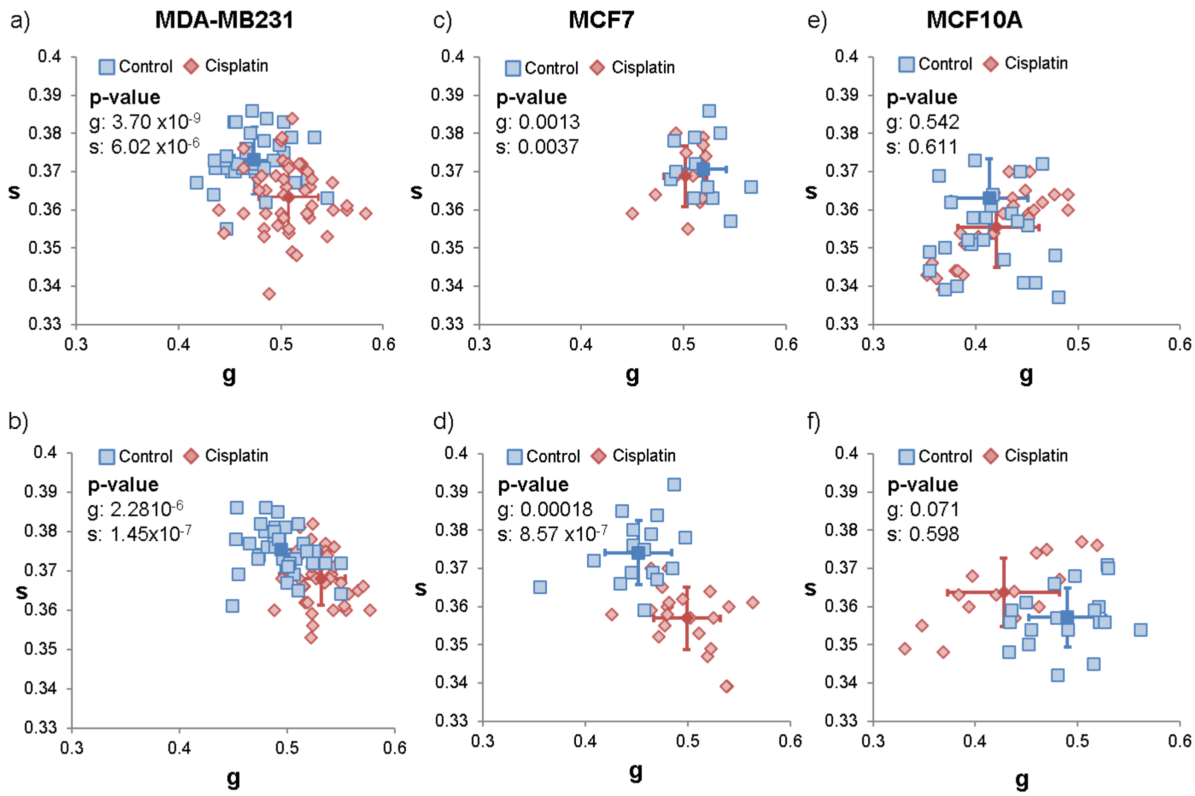

and MCF10A cells with 20mM cisplatin treatment for 4 hours

a) The g and s values of control and cisplatin treated MDA-MB231. The blue squares are from the controls (untreated MDA-MB231), red diamonds are from the cisplatin treated MDA-MB231, and the solid shapes (square and diamond) with the error bars correspond to the average and the standard deviation of each group (student t-test p values compare the treated group to the control group: g= 3.70241E-09, s= 6.02E-06, free to bound NADH ratio=6.80902E-09). FLIM plot illustrates a rightward shift from long to short lifetimes. **b)** The g and s values of control and cisplatin treated MDA-MB231. was obtained from the student t-test p values compare the treated group to the control group: g= 2.28806E-06, s= 1.45E-07, free to bound NADH ratio= 2.03804E-10). FLIM plot illustrates a rightward shift from long to short lifetimes. **c)** The g and s values of control and cisplatin treated MCF7. The blue squares are from the controls (untreated MCF7), red diamonds are from the cisplatin treated MCF7, and the solid shapes (square and diamond) with the error bars correspond to the average and the standard deviation of each group (student t-test p values compare the treated group to the control group: g= 0.001308, s= 0.0037258, free to bound NADH ratio= 1.57E-06). FLIM plot indicates a leftward shift from short to long lifetimes. **d)** The g and s values of control and cisplatin treated MCF7. The student t-test p values compare the treated group to the control group: g= 0.000183, s= 8.57E-07, free to bound NADH ratio=9.55475E-08). FLIM plot indicates a leftward shift from short to long lifetimes. **e)** The g and s values of control and cisplatin treated MCF10A. The blue squares are from the controls (untreated MCF10A), red diamonds are from the cisplatin treated MCF10A,

and the solid shapes (square and diamond) with the error bars correspond to the average and the standard deviation of each group (student t-test p values: g= 0.542635, s= 0.611355, free to bound NADH ratio= 0.000154654). FLIM plot illustrates there is no significant shift. **f)** The g and s values of control and cisplatin treated MCF10A. The standard deviation of each group was obtained from thestudent t-test p values: g= 0.071252, s= 0.598522, free to bound NADH ratio= 0.328814). FLIM plot illustrates there is no significant shift.

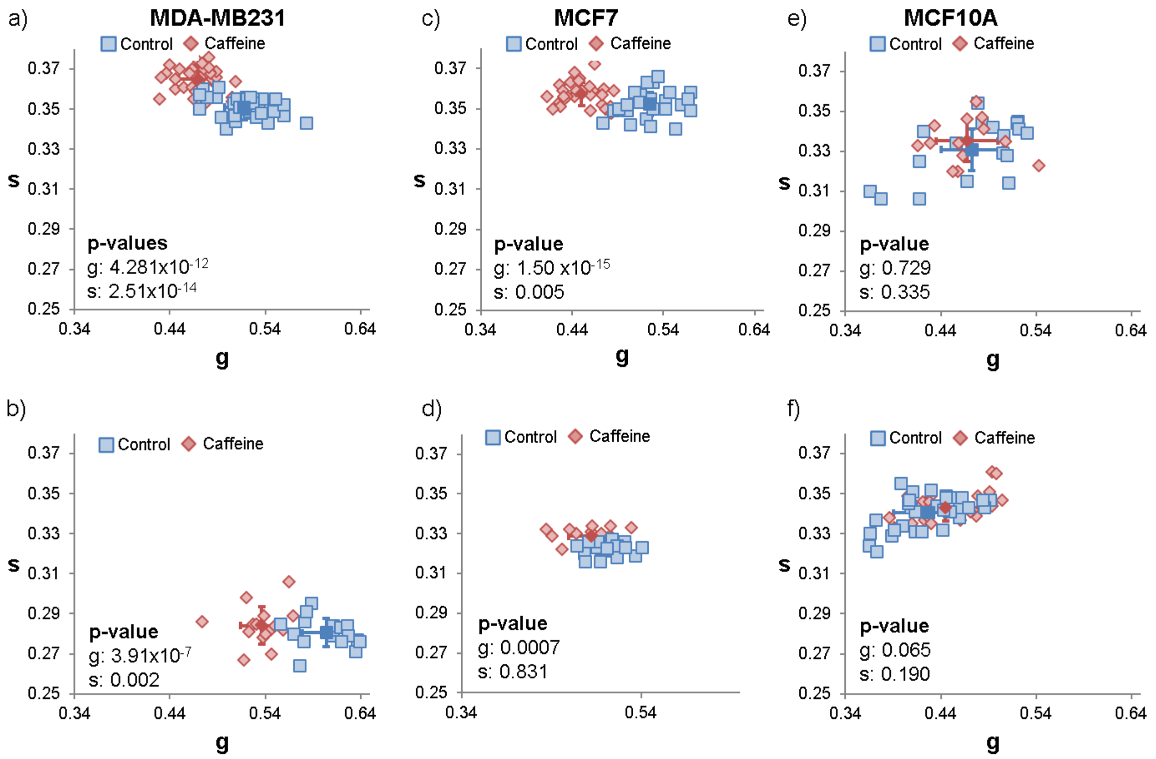

Figure S2. Fluorescence lifetime trajectories of the metabolic state of MDA-MB231, MCF7 and MCF10A cells with 125nM caffeine for 1 hour

a) The g and s values of control and caffeine treated MDA-MB231. The blue squares are from the controls (untreated MDA-MB231), red diamonds are from the caffeine treated MDA-MB231, and the solid shapes (square and diamond) with the error bars correspond to the average and the standard deviation of each group (student t-test p values compare the treated group to the control group: g= 4.28172E-12, s=2.51E-14, free to bound NADH ratio = 3.44489E-15). FLIM plot illustrates a leftward shift from short to long lifetimes **b)** The g and s values of control and caffeine treated MDA-MB231. The error bars correspond to the average and the standard deviation of each group (student t-test p values compare the treated group to the control group: g= 3.91E-07, s= 0.943695, free to bound NADH ratio= 8.52971E-08). FLIM plot illustrates a leftward shift from short to long lifetimes. **c)** The g and s values of control and caffeine treated MCF7. The blue squares are from the controls (untreated MCF7), red diamonds are from the caffeine treated MCF7, and the solid shapes (square and diamond) with the error bars correspond to the average and standard deviation of each group (student t-test p values compare the treated group to the control group: g= 1.50331E-15, s= 0.005623, free to bound NADH ratio= 2.93686E-15). FLIM plot indicates a leftward shift from short to long lifetimes. **d)** The g and s values of control and caffeine treated MCF7. The error bars correspond to the average and standard deviation of each group (student t-test p values compare the treated group to the control group: g= 0.000779, s= 0.831149, free to bound NADH ratio= 0.004614474). FLIM plot indicates a leftward shift from short to long

lifetimes. **e)** The g and s values of control and cisplatin treated MCF10A. The blue squares are from the controls (untreated MCF10A), red diamonds are from the cisplatin treated MCF10A, and the solid shapes (square and diamond) with the error bars correspond to the average and standard deviation of each group (student t-test p values compare the treated group to the control group: g= 0.729926, s= 0.335349, free to bound NADH ratio= 0.46512534). FLIM plot illustrates there is no significant shift. **f)** The g and s values of control and cisplatin treated MCF10A. The error bars correspond to the average and standard deviation of each group (student t-test p values compare the treated group to the control group: g= 0.065807, s= 0.190147, free to bound NADH ratio= 0.096082579). FLIM plot illustrates there is no significant shift.

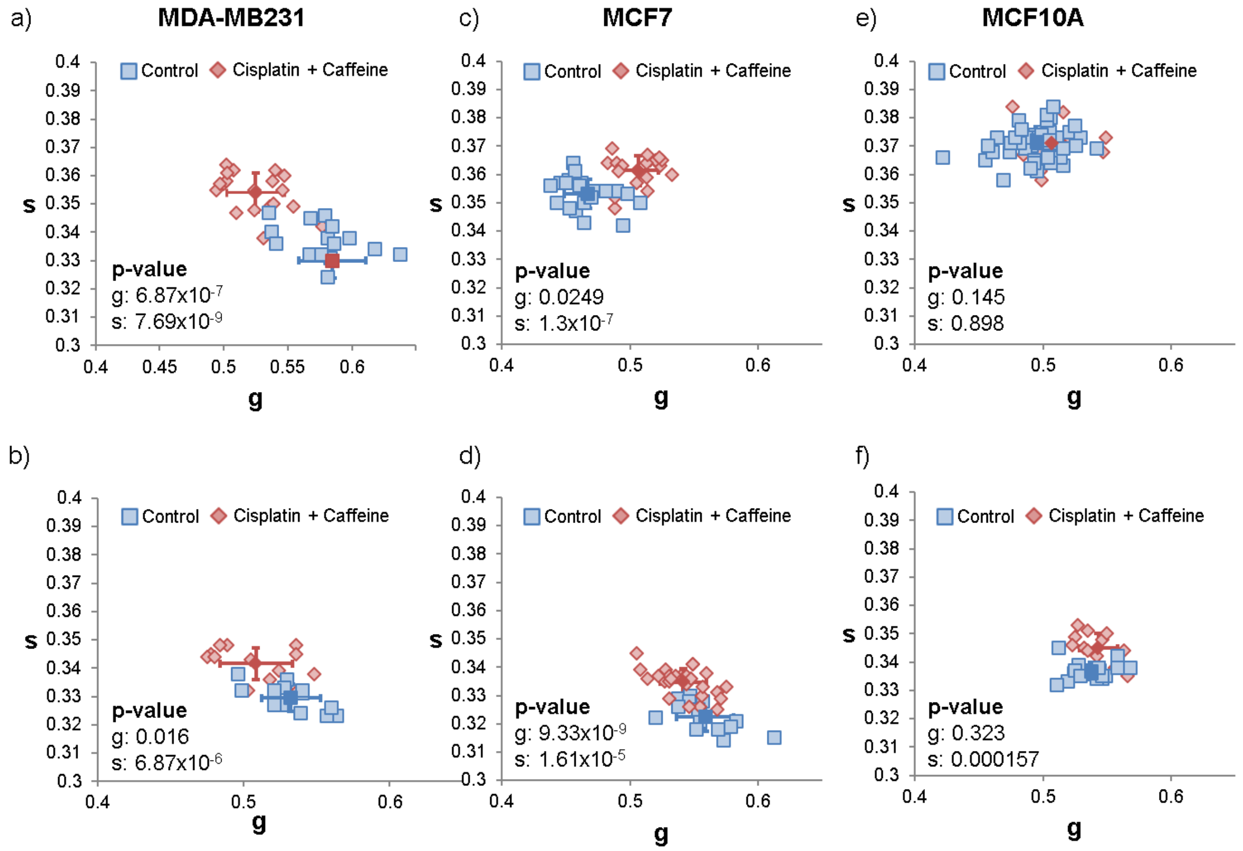

Figure S3. Fluorescence lifetime trajectories display the metabolic state of MDA-MB231, MCF7 and MCF10A cells with 125nM caffeine for 1 hour

a) The g and s values of control and caffeine treated MDA-MB231. The blue squares are from the controls (untreated MDA-MB231), red diamonds are from the caffeine treated MDA-MB231, and the solid shapes (square and diamond) with the error bars correspond to the average and the standard deviation of each group (student t-test p values compare the treated group to the control group: g= 6.87E-07, s= 7.69E-09, free to bound NADH ratio= 1.6159E-08). FLIM plot illustrates a leftward shift from short to long lifetimes. **b)** The g and s values of control and caffeine treated MDA-MB231. The solid shapes (square and diamond) with the error bars correspond to the average and the standard deviation of each group (student t-test p values compare the treated group to the control group: g= 0.016574, s= 8.22E-06, free to bound NADH ratio= 0.000209). FLIM plot illustrates a leftward shift from short to long lifetimes. **c)** The g and s values of control and caffeine treated MCF7. The blue squares are from the controls (untreated MCF7), red diamonds are from the caffeine treated MCF7, and the solid shapes (square and diamond) with the error bars correspond to the average and standard deviation of each group (student t-test p values compare the treated group to the control group: g= 0.024993363, s= 1.35E-07, free to bound NADH ratio=0.000234). FLIM plot indicates a leftward shift from short to long lifetimes. **d)** The g and s values of control and caffeine treated MCF7. The solid shapes (square and diamond) with the error bars correspond to the average and standard deviation of each group (student t-test p values compare the treated group to the control group: g=

9.33991E-09, s= 1.61E-05, free to bound NADH ratio= 0.02896). FLIM plot indicates a leftward shift from short to long lifetimes. **e)** The g and s values of control and cisplatin treated MCF10A. The blue squares are from the controls (untreated MCF10A), red diamonds are from the cisplatin treated MCF10A, and the solid shapes (square and diamond) with the error bars correspond to the average and standard deviation of each group (student t-test p values compare the treated group to the control group: g= 0.14595, s= 0.898326, free to bound NADH ratio=0.1906). FLIM plot illustrates there is no significant shift. **f)** The g and s values of control and cisplatin treated MCF10A. The solid shapes (square and diamond) with the error bars correspond to the average and standard deviation of each group (student t-test p values compare the treated group to the control group: g=0.323458, s= 0.000157, free to bound NADH ratio= 0.0951). FLIM plot illustrates there is no significant shift.
